# Effects of probiotics on liver function, inflammation, and gut microbiota in alcoholic liver injury: a systematic review and meta-analysis

**DOI:** 10.3389/fnut.2025.1717393

**Published:** 2025-12-19

**Authors:** Mohamed J. Saadh, Zahraa Sabah Ghnim, Morug Salih Mahdi, Vimal Arora, M. M. Rekha, Ashish Sharma, Bhanu Juneja, Zafar Aminov, Waam Mohammed Taher, Mariem Alwan, Mahmood Jasem Jawad, Atheer Khdyair Hamad

**Affiliations:** 1 Faculty of Pharmacy, Middle East University, Amman, Jordan; 2 College of Pharmacy, Alnoor University, Nineveh, Iraq; 3 College of MLT, Ahl Al Bayt University, Karbala, Iraq; 4 University Institute of Pharma Sciences, Chandigarh University Mohali, Mohali, Punjab, India; 5 Department of Chemistry and Biochemistry, School of Sciences, JAIN (Deemed to be University), Bangalore, Karnataka, India; 6 Department of Pharmacology, NIMS Institute of Pharmacy, NIMS University Rajasthan, Jaipur, India; 7 Centre for Research Impact & Outcome, Chitkara University Institute of Engineering and Technology, Chitkara University, Rajpura, Punjab, India; 8 Department of Public Health and Healthcare Management, Samarkand State Medical University, Samarkand, Uzbekistan; 9 College of Nursing, National University of Science and Technology, Nasiriyah, Dhi Qar, Iraq; 10 Pharmacy College, Al-Farahidi University, Baghdad, Iraq; 11 Department of Pharmacy, Al-Zahrawi University College, Karbala, Iraq; 12 Gilgamesh Ahliya University, Baghdad, Iraq

**Keywords:** alcohol, inflammation, liver, meta-analysis, probiotic

## Abstract

**Purpose:**

This systematic review and meta-analysis aimed to evaluate the effects of probiotic supplementation on alcohol metabolism, liver function biomarkers, inflammatory indicators, and gut microbiota composition in patients with alcoholic liver disease (ALD), providing insights into their potential therapeutic role.

**Methods:**

A comprehensive search of the PubMed, Scopus, Web of Science, and Cochrane Library databases identified clinical studies assessing probiotic interventions in adults with ALD.

**Results:**

A total of 12 clinical trials conducted between 2008 and 2025 were included. Probiotic supplementation resulted in significant reductions in liver enzymes, including ALT (WMD = −10.10; 95% CI: −15.34, −4.87) and AST (WMD = −13.05; 95% CI: −21.33, −4.78). No significant effects were observed for GGT or ALP. Probiotics did not significantly influence blood alcohol or acetaldehyde levels. Regarding inflammatory markers, probiotics did not significantly affect LPS, TNF-*α*, IL-1β, or IL-6, and IL-10. Microbial analyses showed an increase in beneficial gut bacteria, including *Lactobacillus*, *Bifidobacterium*, *Faecalibacterium*, and *Prevotella*, and a decrease in pathogenic taxa such as *Escherichia* and *Shigella*.

**Conclusion:**

Probiotic supplementation shows promising benefits for improving liver enzyme profiles and modulating the gut microbiota in patients with ALD. However, inconsistent effects on markers of inflammation and alcohol metabolism highlight the need for large-scale, high-quality randomized trials to confirm the therapeutic potential of probiotics in ALD.

## Introduction

Global alcohol consumption has risen significantly, contributing to over 2.6 million deaths annually in 2019, approximately 4.7% of all global mortality (1). Young adults are particularly impacted; for instance, 13% of alcohol-attributable deaths occur among individuals aged 20 to 39 (2). Alcoholic liver disease (ALD) is a major cause of chronic liver injury and is responsible for nearly half of all liver-related deaths worldwide (3). These patterns often reflect broader socioeconomic shifts, including increased alcohol availability driven by economic growth and changing social norms (4).

Seminal studies first identified elevated levels of bacterial endotoxin (lipopolysaccharide, LPS) in the portal circulation of patients with ALD, implicating intestinal barrier dysfunction in the pathogenesis of liver inflammation (5). Chronic ethanol exposure disrupts gut–liver homeostasis, permitting translocation of microbial products that activate hepatic immune responses (6).

The gut–liver axis plays a pivotal role in maintaining liver health, and chronic alcohol intake significantly impairs this balance (7). Alcohol abuse causes an imbalance in the gut microbiome, characterized by a decrease in beneficial bacteria and an increase in harmful organisms (8). Specifically, ALD is consistently linked to a reduction in anti-inflammatory, short-chain fatty acid (SCFA)–producing bacteria such as *Faecalibacterium, Roseburia, Ruminococcus*, and other members of the *Firmicutes* and *Bacteroidetes* phyla (9, 10). On the other hand, there is an overgrowth of potentially harmful *Enterobacteriaceae*, including *Escherichia, Shigella*, and *Klebsiella*, as well as a higher abundance of *Enterococcus* (10, 11). These microbial shifts reduce overall gut diversity and compromise the integrity of the mucosal barrier.

Probiotic supplements have shown therapeutic promise in treating ALD by influencing the gut–liver axis (12). Multiple strains have been studied in both laboratory and early clinical trials. Notably, *Lactobacillus* strains, such as *L. rhamnosus GG, L. casei, L. plantarum,* and *L. helveticus*, along with *Bifidobacterium* strains like *B. bifidum*, and spore-forming species like *Clostridium butyricum*, have been studied (10–12). These probiotics help restore gut balance disrupted by alcohol by increasing beneficial bacteria such as *Lactobacilli and Bifidobacteria,* while reducing harmful gram-negative bacteria like *Escherichia coli* (13). Meta-analyses indicate that probiotic supplementation can reduce intestinal endotoxemia, restore microbial balance, and attenuate hepatic inflammation (14, 15). Collectively, these effects support their utility as a promising adjunct therapy for ALD.

To provide a comprehensive synthesis of clinical evidence on the efficacy of probiotics in ALD, with particular focus on their impacts on alcohol metabolic parameters, hepatic function biomarkers, systemic inflammation, and gut microbiota alterations. This study aims to systematically evaluate the effects of probiotic supplementation on alcohol metabolism parameters, hepatic function, inflammatory markers, and gut microbiota profiles in individuals with ALD through a meta-analysis of clinical trials. By integrating these methodologies, this review seeks to provide comprehensive insights into the therapeutic potential of probiotics in ALD and identify priorities for future clinical research.

## Methods

### Study design

The study was conducted as a systematic review and meta-analysis following the Preferred Reporting Items for Systematic Reviews and Meta-Analyses (PRISMA) guidelines (16).

### Eligibility criteria

Clinical trials evaluating the effects of probiotics supplementation on alcoholic liver injury or alcoholic liver disease were included. Observational studies, review articles, and non-randomized trials were excluded. The PICO framework was used to determine the inclusion criteria. Participants (P) of patients who were newly diagnosed with alcoholic liver injury or alcoholic liver disease of both genders were included. The intervention (I) was probiotics supplementation at any dosage and duration. Comparators (C) included a placebo, no intervention. Outcomes (O) were changes in alcohol metabolism, liver function, inflammatory biomarkers, and gut microbiota.

### Search strategy

A comprehensive search was conducted in electronic databases, including PubMed, Scopus, Web of Science, and Cochrane Library, from inception to October 2025. The search strategy combined keywords and Medical Subject Headings (MeSH) related to “probiotics,” “alcoholic liver disease,” and “alcohol metabolism.” The full search strategy is provided in Supplementary File. No language restrictions were applied.

### Study selection

Search records were uploaded into a reference manager and de-duplicated. Two reviewers independently screened titles and abstracts to identify potentially eligible studies. Full-text articles were then evaluated against the predefined inclusion criteria, with any disagreements resolved through consultation with a third reviewer. The study selection process was reported using a PRISMA flow diagram.

### Data extraction

Two reviewers independently extracted data using a standardized form, recording key information such as study characteristics (author, publication year, location, and design), participant details (sample size and age), intervention features (probiotic dose, duration, and strains), and outcome measures (mean ± standard deviation [SD] changes). Any differences between reviewers were resolved by consensus.

### Risk of bias assessment

The methodological quality of included studies was assessed using the Cochrane Risk of Bias tool (17). Each domain was rated as “low,” “unclear,” or “high risk” of bias. Two reviewers independently conducted the assessments, and any disagreements were resolved through discussion with a third reviewer.

### Statistical analysis

Statistical analyses were performed using Stata Statistical Software, version 14 (StataCorp, College Station, TX, USA), with a random-effects model employed to pool the data. Weighted mean differences (WMDs) and their corresponding 95% confidence intervals (CIs) were calculated to estimate the overall effect sizes (18). Heterogeneity among studies was assessed using the *I*^2^ statistic, with values greater than 50% indicating substantial heterogeneity (19). Sensitivity analyses were conducted using the leave-one-out method to evaluate the influence of individual studies on the pooled results (20). Publication bias was assessed visually through funnel plots and statistically using Begg’s and Egger’s tests. In cases of detected publication bias, trim-and-fill analysis was applied. Statistical significance was defined as a *p*-value < 0.05 for all analyses.

## Results

### Characteristics of included studies

**Figure 1:**
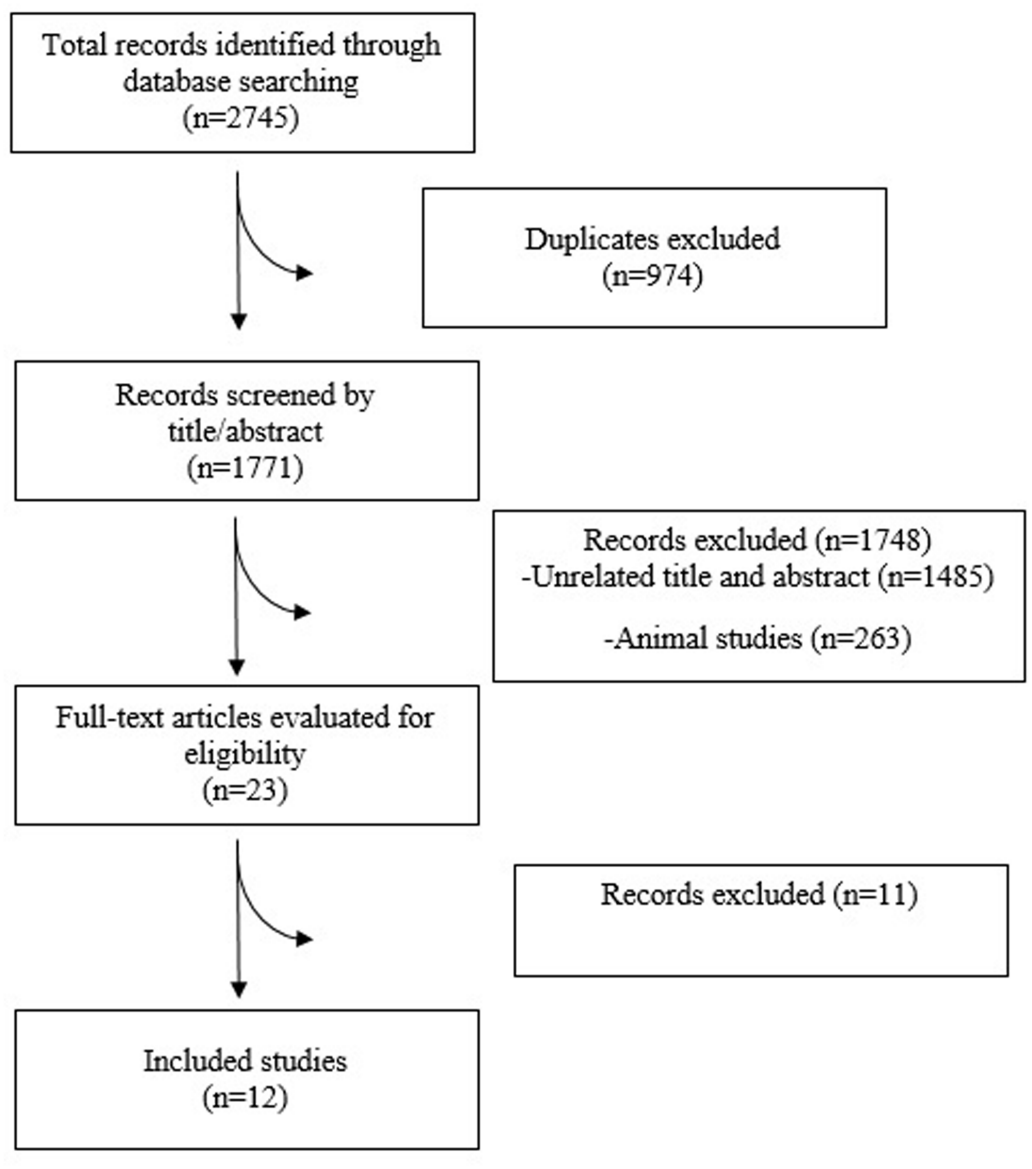
Flow diagram of study selection.

The PRISMA flowchart (Figure 1) systematically illustrates the study selection process. Out of 2,745 records initially identified, 974 duplicates were removed, and 1,748 studies were excluded after title and abstract screening. Ultimately, 23 full-text articles were assessed for eligibility. A total of 12 clinical trials conducted between 2008 and 2025 were included, covering participant groups from Korea, China, Russia, Japan, the United States, and the United Kingdom (10–12, 21–29) (Table 1). Sample sizes ranged from 20 to 158 individuals, and the distribution of participants across probiotic and control groups varied by study design. Participants consisted of adults aged 18 to 65 years, including both males and females (with males comprising 55–75%), exhibiting disease severity from mild to moderate ALD; a limited number of studies encompassed individuals with more advanced liver injury. The studies investigated a broad spectrum of probiotic preparations, including *Lacticaseibacillus rhamnosus R0011, Lactobacillus helveticus R005, Bifidobacterium bifidum, Lactobacillus plantarum 8PA3, Weizmannia coagulans BC99, Lactobacillus casei Shirota, Lactobacillus gasseri, Bifidobacterium lactis, Bifidobacterium breve*, and various multi-strain formulations. Intervention periods ranged from 1 to 8 weeks, representing both short-term and mid-term probiotic administration. Across the included trials, commonly reported outcomes comprised liver enzyme markers: ALT, AST, GGT, and ALP, and inflammatory biomarkers such as TNF-*α*, IL-6, IL-10, IL-1β, as well as lipopolysaccharide (LPS). In addition, several studies assessed enzymes involved in alcohol metabolism, specifically alcohol dehydrogenase (ADH) and aldehyde dehydrogenase (ALDH).

**Table 1:** Basic characteristics of included studies.

Author (year)	Country	Sample size	Probiotics/Control	Age	Disease severity	Interventions	Duration (weeks)	Outcome
Stadlbauer et al. (23)	U. K	20	12/8	53.5	Mild ALD	*Lactobacillus casei Shirota* 6.5 × 10^9^ CFU, 3 times/day	4 weeks	ALT
Kirpich et al. (12)	Russia	66	32/34	>18y	Mild–moderate ALD	*Bifidobacterium. bifidum 0.9 × 10^8^ CFU + Lactobacillus. plantarum 0.9 × 10^9^ CFU*	1 week	ALT, AST, GGT
Zhang et al. (24)	China	66	32/34	46	Mild ALD	*Bifidobacterium,lactob-acillus*	1 week	ALT, AST, GGT
Koga et al. (25)	Japan	37	18/19	53.5	Mild–moderate ALD	*Lactobacillus casei Shirota YIT 9029* Twice daily (dose per bottle depends on product)	4 weeks	ALT, AST, GGT, TNF-α, IL-6
Han et al. (26)	Korea	117	60/57	>20y	Mild–moderate ALD	*Lactobacillus subtilis and streptococcus faecium* 1,500 mg/day	1 week	ALT, AST, GGT, ALP, LPS, TNF-α, IL-1β
Zhu et al. (27)	China	80	40/40	47	Mild ALD	*Polyene phosphatidylcholine capsules+* *Clostridium Butyricum Tablets* *0.7 g/day tid*	8 weeks	ALT, AST, TNF-α, IL-6
Li et al. (22)	China	158	54/46	30-65y	Mild–moderate ALD	*Lactobacillus casei strain shirota (LcS)* Low dose: 100 mL/day; High dose: 200 mL/day (fermented milk)	8 weeks	ALT, AST, GGT, TNF-α, IL-6, IL-10, IL-1β
Jung et al. (21)	Korea	54	54/54	19-65y	Mild–moderate ALD	*Duolac ProAP4 (Lactobacillus gasseri CBT LGA1, L casei CBT LC5, Bifidobacterium lactis CBT BL3, Bifidobacterium breve CBT BR3)* 4 capsules/day; total ≈5 × 10^8^ CFU/day	8 weeks	ALT, AST, GGT, ADH, ALDH
Gupta et al. (28)	Korea	89	44/45	>20y	Mild ALD	*Lacticaseibacillus rhamnosus* R0011 and *Lactobacillus helveticus* R005 120 mg/day	1 week	ALT, AST, GGT, ALP
Vatsalya et al. (29)	USA	46	24/22	44.5	Mild–moderate ALD	*Lactobacillus rhamnosus GG*	4 weeks	ALT, AST
Sun et al. (11)	Korea	26	26/26	19-40y	Mild ALD	*Wilac L probiotic complex (Levilactobacillus brevis WiKim 0168, Leuconostoc mesenteroides WiKim 0172)* 1 × 10^9^ CFU (single dose), capsule	3–5 weeks	ADH, ALDH, ALT, AST
Zhang et al. (10)	China	60	30/30	18-65y	Mild–moderate ALD	*Weizmannia coagulans BC99* 3 g/day (≈1 × 10^10^ CFU), powder/capsule	8 weeks	ALT, AST, GGT, LPS, TNF-α, IL-6, IL-10

### Risk of bias assessment

In this analysis, most studies showed a low risk of bias for incomplete outcome data and selective reporting. Still, many had unclear or high risk regarding random sequence generation and blinding of participants or outcome assessors. Overall, the methodological quality varied, with some studies showing notable limitations in design and execution (Table 2). Overall, while the methodological rigor was acceptable in most trials, the notable sources of bias highlight the need for cautious interpretation of pooled results.

**Table 2:** Quality of included studies in the meta-analysis.

Author (year)	Random sequence generation	Allocation concealment	Blinding of participants & personnel	Blinding of outcome assessment	Incomplete outcome data	Selective outcome reporting	Other sources of bias
Stadlbauer et al. (23)	U	U	H	H	L	L	L
Kirpich et al. (12)	H	U	H	L	L	L	L
Zhang et al. (24)	L	U	L	H	L	L	L
Koga et al. (25)	H	U	L	H	L	L	L
Han et al. (26)	L	U	L	H	L	L	L
Zhu et al. (27)	L	U	U	U	L	L	L
Li et al. (22)	L	U	L	H	L	L	L
Jung et al. (21)	L	U	L	U	L	L	L
Gupta et al. (28)	L	L	L	H	L	L	L
Vatsalya et al. (29)	L	U	L	L	L	L	L
Sun et al. (11)	L	L	L	L	L	L	L
Zhang et al. (10)	L	H	L	L	L	L	L

Footprint: H, high risk of bias; L, low risk of bias; U, unclear risk of bias.

### Alcohol metabolism

**Figure 2:**
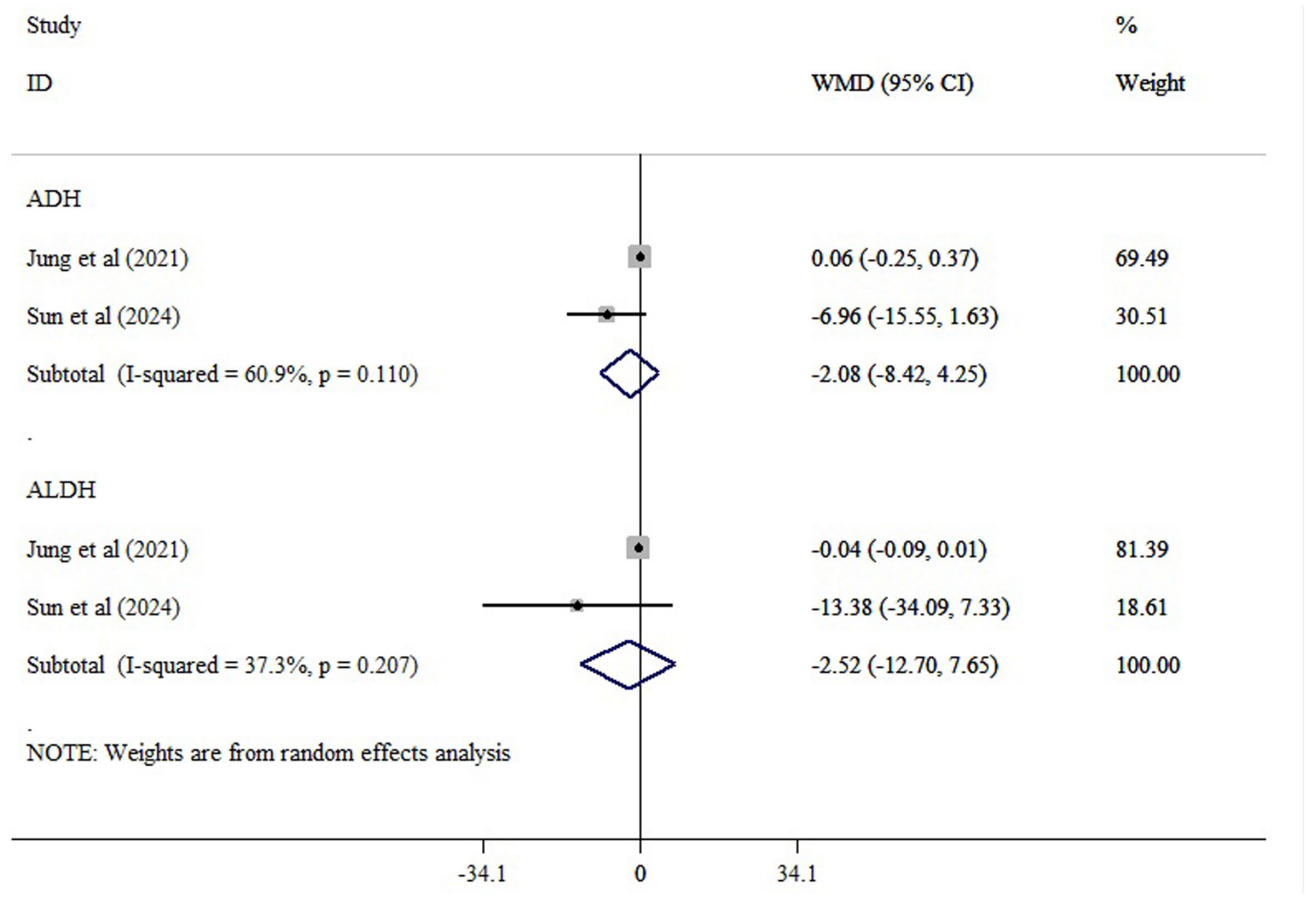
The effects of probiotic supplementation on ADH, and ALDH.

Two studies evaluated the impact of probiotics on blood alcohol concentrations (ADH activity) and blood acetaldehyde levels (ALDH activity) in patients with alcoholic liver disease. The meta-analysis detected no significant pooled effects for either biomarker: blood alcohol (WMD = −2.08; 95% CI: −8.42 to 4.25; *p* = 0.520) or acetaldehyde (WMD = 2.52; 95% CI: −12.70 to 7.65; *p* = 0.627) (Figure 2). Heterogeneity was moderate for blood alcohol (*I*^2^ = 60.9%) and low for acetaldehyde (*I*^2^ = 37.3%). Although statistically non-significant, all included studies demonstrated a consistent reduction in these biomarkers following probiotic supplementation. The consistent reduction across studies suggests a potential metabolic effect of probiotics, warranting confirmation through larger, methodologically robust clinical trials.

### Liver function parameters

**Figure 3:**
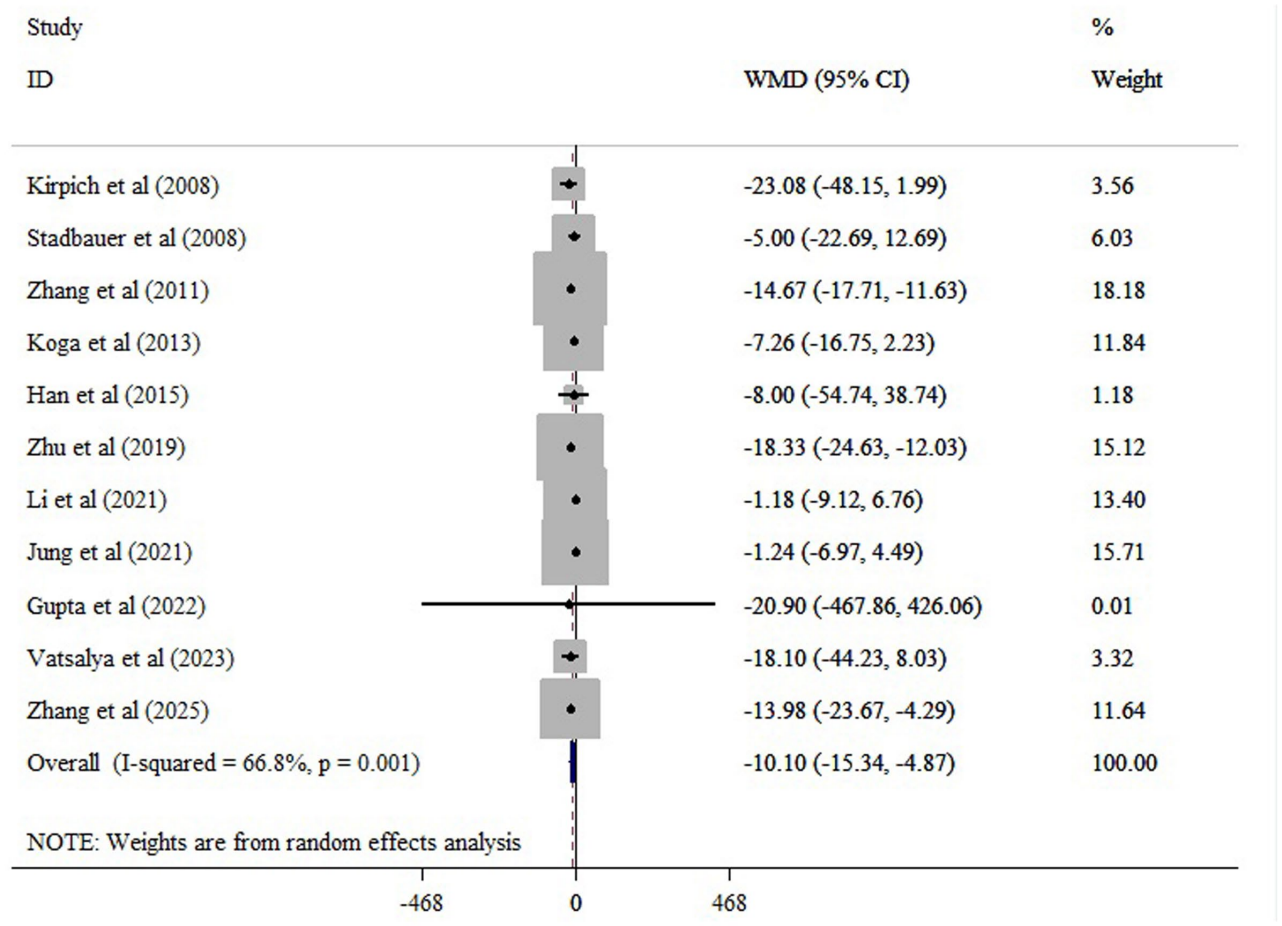
The effects of probiotic supplementation on ALT.

**Figure 4:**
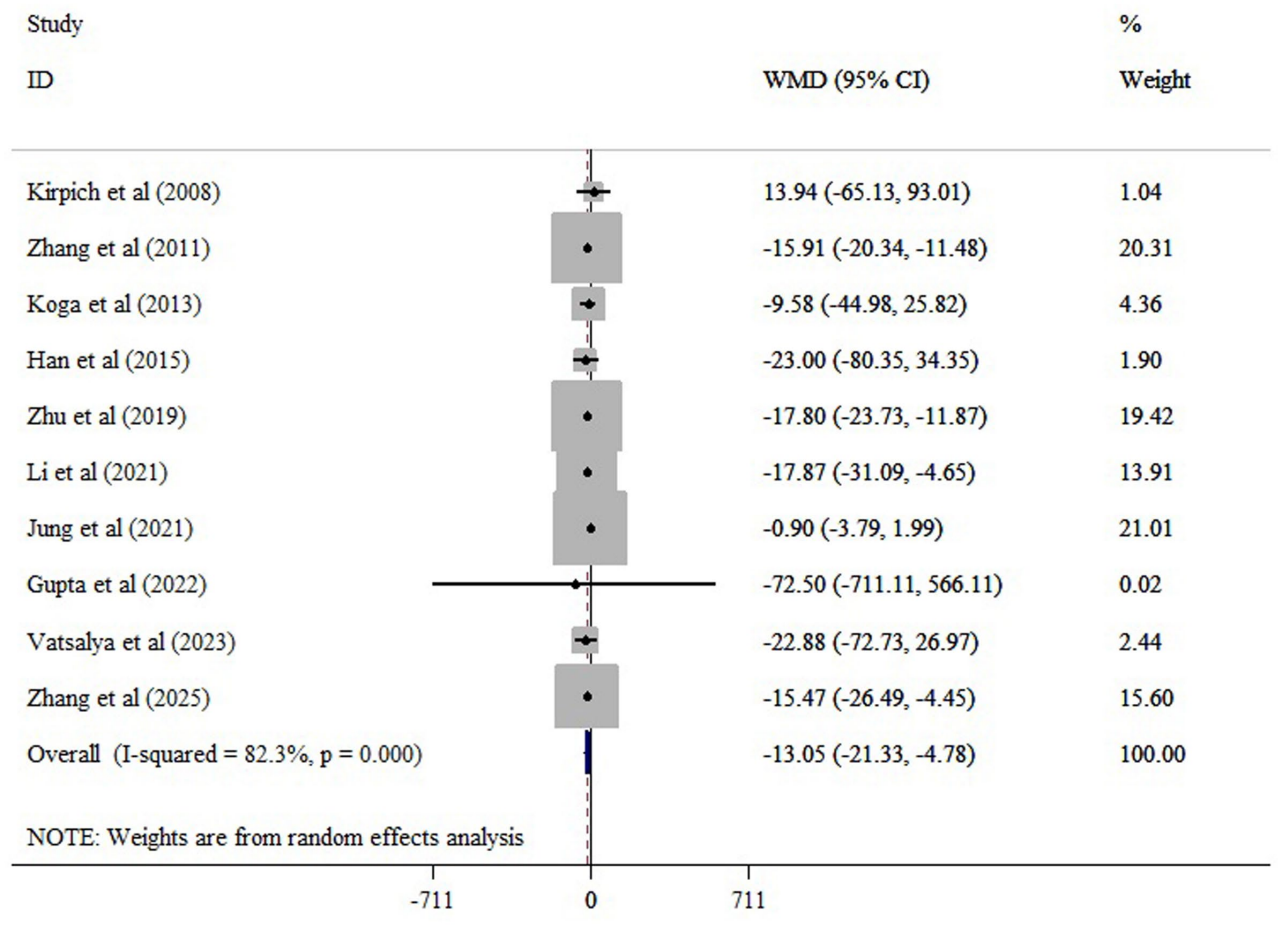
The effects of probiotic supplementation on AST.

**Figure 5:**
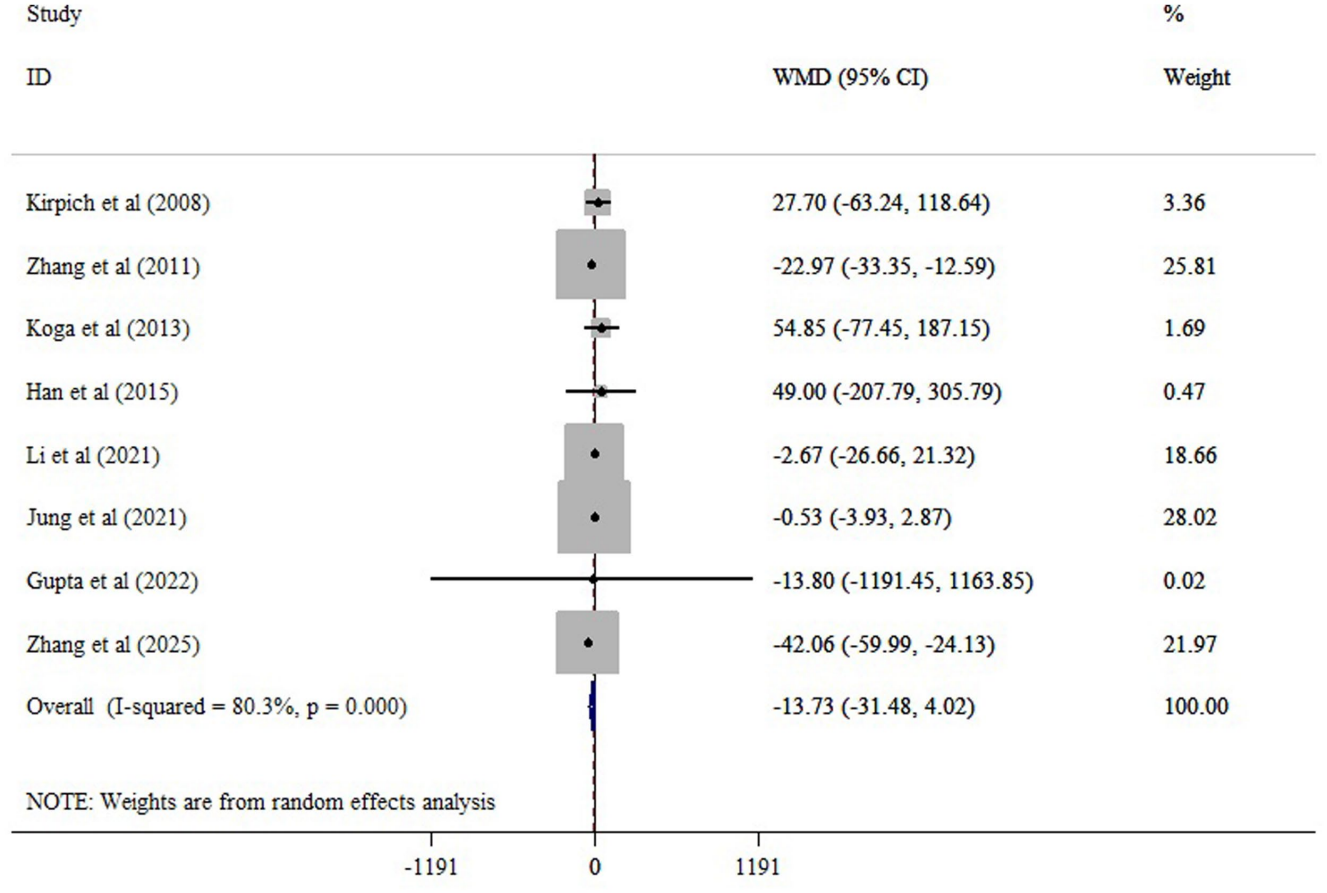
The effects of probiotic supplementation on GGT.

**Figure 6:**
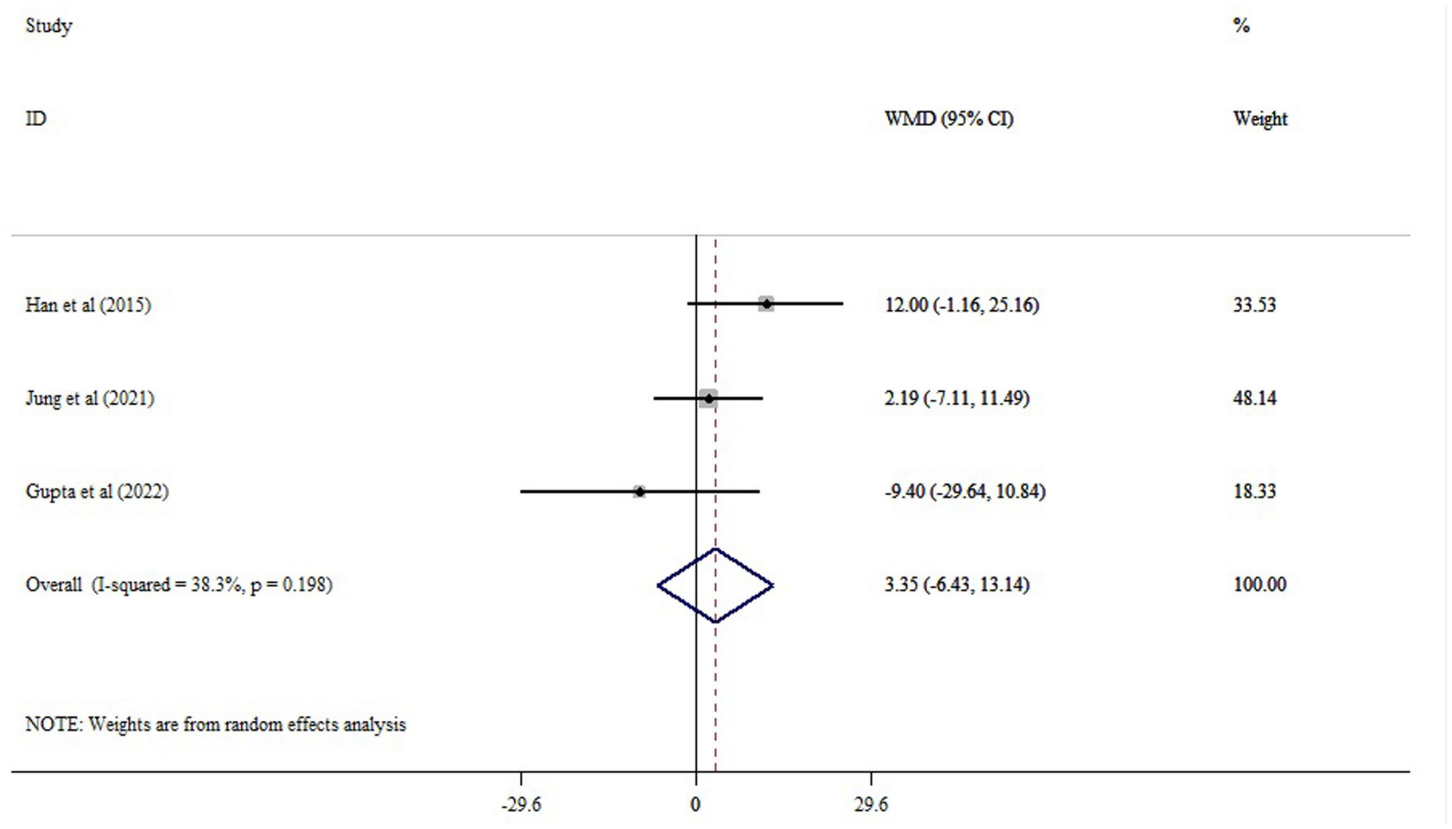
The effects of probiotic supplementation on ALP.

The meta-analysis of 11 comparisons revealed that probiotic supplementation significantly reduced ALT (WMD = −10.10; 95% CI: −15.34, −4.87; *p* < 0.001), with moderate heterogeneity (*I*^2^ = 66.8%; *p* < 0.001) (Figure 3). Probiotics supplementation significantly reduced AST (10 studies), with a pooled WMD of −13.05 (95% CI: −21.33, −4.78; *p* < 0.001) and substantial heterogeneity (*I^2^* = 82.3%, *p* < 0.001) (Figure 4). Also, probiotics did not have a significant effect on GGT (WMD = −13.73; 95% CI: −31.48, 4.02; *p* = 0.130; *I^2^* = 80.3%, *p* < 0.001) (Figure 5), and ALP (WMD = 3.35; 95% CI: −6.43, 13.14; *p* = 0.501; *I^2^* = 38.3%, *p* = 0.198) (Figure 6).

Sensitivity analysis confirmed the robustness of our findings on ALT and AST (*p* < 0.05). Sensitivity analysis showed that excluding the Jung et al. study (21) substantially changed the estimated effect of probiotics on GGT (WMD = −21.14; 95% CI: −37.23, −5.05; *p* < 0.05). Begg’s and Egger’s tests did not indicate bias due to small study effects (*p* > 0.05). Asymmetry was observed in the funnel plot, possibly due to publication bias for GGT (Supplementary Figures 1–3). However, the trim-and-fill analysis yields a similar result to the overall result (WMD = −16.74; 95% CI: −34.05, 0.55; *p* ˃ 0.05) (Supplementary Figure 4).

### Inflammatory biomarkers

**Figure 7:**
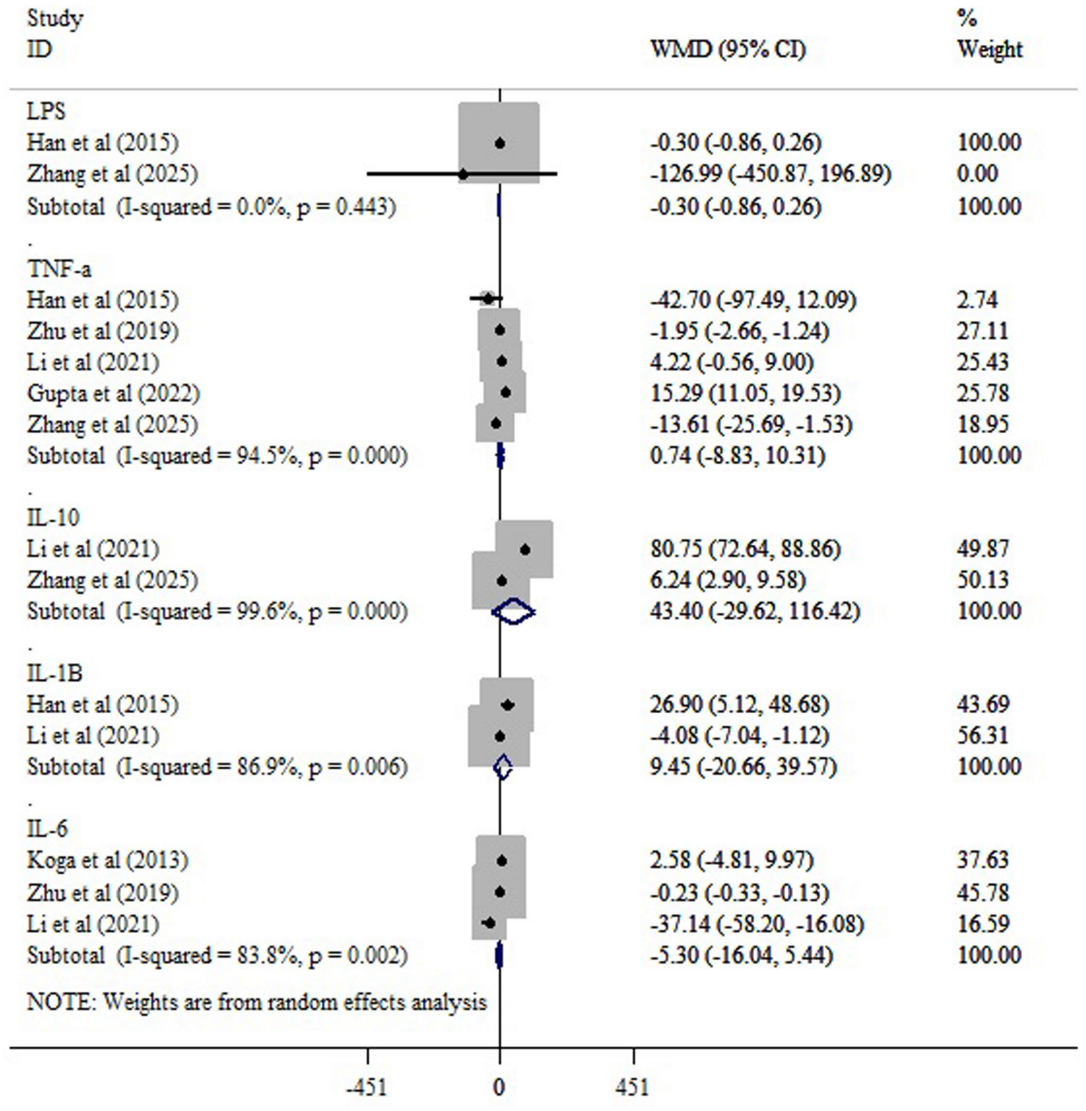
The effects of probiotic supplementation on LPS, TNF-a, IL-, IL-10 and IL-6.

Our finding revealed that probiotics had no considerable impact on LPS (WMD = −0.30; 95% CI: −0.86, 0.26; *p* = 0.720; *I*^2^ = 0.0%, *p* = 0.443), TNF-a (WMD = 0.74; 95% CI: −8.83, 10.31; *p* = 0.182; *I*^2^ = 94.5%, *p* < 0.001), IL-1B (WMD = 9.45; 95% CI: −20.66, 39.57; *p* = 0.406; *I*^2^ = 86.9%, *p* < 0.001), and IL-6 (WMD = −5.30; 95% CI: −16.04, 5.44; *p* = 0.428; *I*^2^ = 83.8%, *p* = 0.002) (Figure 7). The meta-analysis showed that probiotic supplementation did not lead to a statistically significant increase in IL-10 levels compared with the control group (WMD = 43.40; 95% CI: −29.62 to 116.42; *p* = 0.242; *I*^2^ = 99.6%, *p* < 0.001) (Figure 7).

### Microbial analysis

Clinical studies (Table 3) show that probiotic supplementation in ALD enriches beneficial bacteria (*Lactobacillus, Bifidobacterium, Faecalibacterium, Prevotella, Roseburia*) and reduces potentially pathogenic taxa (*Escherichia, Shigella, Klebsiella, Enterococci, Proteobacteria, Fusobacteria*), improves gut microbial balance, and may affect both alpha and beta diversity, suggesting potential therapeutic benefits.

**Table 3:** Changes in intestinal flora after probiotic supplementation.

Study	Probiotic type	Changes in intestinal microbiota
Gupta et al. (28)	Lacticaseibacillus rhamnosus R0011 & *Lactobacillus helveticus* R0052	↑ Bacteroidetes, *Faecalibacterium prausnitzii*, *Veillonella dispar*, Enterobacter spp.; ↓ Proteobacteria, Fusobacteria, *E. coli*, *B. fragilis*, *F. mortiferum*
Han et al. (26)	Lactobacillus subtilis & *Streptococcus faecium*	↓ *Escherichia coli*, Enterococci
Kirpich et al. (12)	*Bifidobacterium bifidum* & *Lactobacillus plantarum* 8PA3	↑ Lactobacillus, Bifidobacteria
Koga et al. (25)	*Lactobacillus casei* Shirota YIT 9029	↑ Obligate anaerobic bacteria; ↓ Enterobacteriaceae
Li et al. (22)	*Lactobacillus casei*	↑ Lactobacillus, Bifidobacterium
Zhang et al. (24)	Bifidobacterium, Lactobacillus, Enterococcus	↑ Bifidobacteria, Lactobacilli, Enterococci
Zhu and Wu (27)	*Clostridium butyricum*	↑ Bifidobacteria, Lactobacilli; ↓ *Escherichia coli*
Zhang et al. (10)	Weizmannia coagulans BC99	↑ Prevotella, Faecalibacterium, Roseburia; ↓ Bacteroides, Escherichia, Shigella, Klebsiella

“↑” indicates increase; “↓” indicates decrease.

## Discussion

Our systematic review and meta-analysis of 12 studies demonstrates that probiotic supplementation offers measurable benefits in ALD. The most robust finding was a significant reduction in hepatic transaminases: pooled weighted mean differences showed probiotics lowered ALT and AST by clinically meaningful margins (30). In contrast, effects on other liver enzymes (GGT) and (ALP) were not statistically significant in the primary analysis (although sensitivity testing suggested a possible GGT reduction after omitting one outlying trial). No significant pooled effects were detected for blood alcohol or acetaldehyde levels (ADH/ALDH activity), LPS, or the pro-inflammatory cytokines TNF-*α*, IL-1β, IL-6, and anti-inflammatory cytokine IL-10. Microbial analyses from the included studies consistently showed enrichment of beneficial taxa (*Lactobacillus, Bifidobacterium, Faecalibacterium*, etc.) and reductions in pathogenic proteobacteria (*Escherichia, Shigella, Klebsiella, Enterococcus*, etc.) (30, 31).

These outcomes largely agree with previous reports. For example, Xiong et al. (30) found that probiotics significantly improved liver enzymes (ALT, AST, GGT) and serum albumin, while also increasing Bifidobacteria and reducing *E. coli* in ALD patients. Our ALT/AST findings mirror theirs, and like them, we saw no significant change in TNF-α or IL-6 (30). The consistency of enzyme improvement across studies strengthens confidence that probiotics have a genuine hepatoprotective effect. Mechanistically, these effects likely stem from modulation of the gut–liver axis: probiotics can restore intestinal barrier function, reduce endotoxin (LPS) leakage, and dampen TLR4-mediated hepatic inflammation (31, 32). For example, Lactobacillus species like LGG have demonstrated the ability to repair alcohol-induced intestinal damage and reduce oxidative stress in the liver (33). Probiotics strengthen tight junctions and increase mucus production, which reduces the translocation of pathogen-associated molecular patterns (PAMPs) and helps prevent excessive activation of the innate immune system in the liver.

Probiotics enhance the gut–liver axis by restoring barrier integrity and diminishing the translocation of microbial products (34). Chronic alcohol consumption damages the intestinal lining, enabling LPS and other toxins to reach the liver and promote inflammation (35). Probiotic supplementation re-establishes tight junctions, boosts SCFA production, and reduces gut permeability (36, 37). As a result, fewer endotoxins reach the portal circulation, potentially reducing TLR4-driven cytokine cascades in hepatocytes and Kupffer cells (38). This mechanism plausibly explains the lowered ALT/AST we observed (31).

Despite these improvements in enzyme activity, it was unexpected that systemic inflammatory markers remained unchanged or even decreased. Preclinical studies suggest probiotics should lower TNF-*α* and IL-6 (31), but our analysis did not find this. One possibility is that circulating cytokine levels do not fully capture local hepatic immune responses. Additionally, the trials varied widely in patient severity, intervention duration, and analytical methods, which may have diluted any signal.

The meta-analysis indicated that probiotic supplementation did not produce a statistically significant increase in IL-10 levels relative to the control group. This finding is somewhat unexpected given that *in vitro* studies consistently show that Bifidobacterium and Lactobacillus strains can induce IL-10 secretion (39). A plausible explanation is that IL-10 may already be upregulated in ALD as part of a compensatory anti-inflammatory mechanism, thereby limiting the observable incremental effect of probiotics. Additionally, heterogeneity in assay timing, measurement techniques, or probiotic strain composition across studies may have contributed to the absence of a detectable change. Collectively, these considerations highlight the need for larger, rigorously standardized clinical trials to more accurately characterize the immunomodulatory influence of probiotic therapy.

It is important to note that several outcomes, including blood alcohol concentrations, LPS levels, and pro-inflammatory cytokines such as TNF-*α* and IL-6, did not show statistically significant improvements with probiotic supplementation. These nonsignificant findings suggest that the therapeutic effects of probiotics in ALD are probably selective rather than widespread. Although our findings indicate a clear improvement in hepatic transaminases and gut microbial composition, the lack of significant changes in systemic inflammatory markers highlights the importance of cautious interpretation regarding the broader therapeutic benefits of probiotics. Variability in disease severity, probiotic strains, intervention durations, and analytic methods may have limited the ability to detect modest biological effects. Therefore, the current evidence suggests that probiotics may offer adjunctive, rather than comprehensive, benefits in ALD, and further standardized, well-powered trials are required to more precisely define their clinical impact.

Our review also emphasized consistent alterations in gut microbiota caused by probiotics. Although a formal meta-analysis of taxa was not possible, all trials showed an increase in beneficial genera and a decrease in pathogens following treatment. For example, several studies observed an increase in Bifidobacterium and a decrease in Escherichia levels with probiotic use (40). These microbial shifts likely play a role in the clinical effects: higher levels of SCFA-producing bacteria (such as Roseburia and Faecalibacterium) may enhance the gut barrier and reduce liver inflammation. This is consistent with other research indicating that probiotics can restore alcohol-related dysbiosis and prevent LPS leakage (41, 42). Although our quantitative results focused on biochemical markers, the consistent microbiome findings strengthen the conclusion that probiotics help restore gut–liver homeostasis in ALD.

Several limitations should be considered carefully. The trials included in the analysis were diverse in their design: they differed in probiotic strains (single or multi-species), doses, and treatment durations, which ranged from 1 to 8 weeks. Most patient populations consisted of adults with mild to moderate ALD, often from Asia, which may limit how applicable the findings are. Numerous studies were limited in size, and several exhibited unclear or substantial risk of bias concerning randomization and blinding procedures. This heterogeneity contributed to elevated I^2^ statistics for certain outcomes (AST, TNF-*α*). Publication bias may exist, as indicated by asymmetry in the funnel plot for GGT, although the trim-and-fill analysis did not significantly alter the conclusion. All outcomes were surrogate biomarkers; no trial reported concrete clinical endpoints like progression to cirrhosis, mortality, or liver histology. Therefore, our results should be considered hypothesis-generating rather than conclusive evidence of clinical benefit.

Looking forward, future studies should standardize probiotic interventions by selecting strains with established gut-barrier or anti-inflammatory benefits and include longer follow-up periods to evaluate long-term effects. Using probiotics together with prebiotics or dietary changes could potentially increase their effectiveness. It will also be helpful to link microbial and metabolomic changes to clinical outcomes. Our meta-analysis offers the most thorough evidence to date that probiotics can help correct liver enzyme irregularities and restore gut balance in ALD. These findings suggest that probiotic therapy could be a useful supplement alongside abstinence and nutritional support. However, more extensive, carefully designed trials are necessary to truly determine its effect on outcomes that matter to patients.

Given the encouraging hepatoprotective signals, research priorities should include: (1) large-scale RCTs focusing on clinically relevant endpoints (liver histology, hospitalization, survival); (2) mechanistic studies in humans to link specific microbial shifts with biochemical improvements; and (3) exploration of personalized probiotics based on individual microbiome profiles. Integrating these approaches will clarify how best to harness the gut–liver axis in treating ALD.

## Conclusion

Probiotic supplementation shows beneficial effects in patients with alcoholic liver disease. It significantly improves liver enzyme profiles, particularly ALT and AST. Probiotics also enhance gut microbiota by increasing beneficial bacteria and reducing pathogenic taxa. Effects on alcohol metabolism and inflammatory markers were inconsistent across studies. Variations in study design, probiotic strains, and intervention duration may explain these discrepancies. Future large-scale, high-quality trials are needed to confirm these therapeutic benefits and their clinical relevance.

## Data Availability

The original contributions presented in the study are included in the article/Supplementary material, further inquiries can be directed to the corresponding author.
