# Supplementary material for: Effects of probiotics on liver function, inflammation, and gut microbiota in alcoholic liver injury: a systematic review and meta-analysis
Source: Front Nutr. 2025 Dec 19;12:1717393. doi: 10.3389/fnut.2025.1717393 (PMC12757394; doi:10.3389/fnut.2025.1717393)
Supplement: Supplementary file 1 [file Data_Sheet_1.docx]

Supplementary Material

# Supplementary Tables

## PubMed database search query

| ( "Liver Diseases, Alcoholic"[Mesh] OR (alcoholic AND (liver disease* OR steatosis OR fibrosis OR hepatitis OR cirrhosis)) AND ( "Probiotics"[Mesh] OR Probiotics[Title/Abstract] OR Probiotic[Title/Abstract]) AND ( random*[Title/Abstract] OR placebo*[Title/Abstract] OR blind*[Title/Abstract] OR "Randomized Controlled Trial"[Publication Type]) |
| --- |

## Web of Science database search query

| TS=((alcoholic AND ("liver disease*" OR steatosis OR fibrosis OR hepatitis OR cirrhosis)) AND (probiotics OR probiotic) AND (random* OR placebo* OR blind*)) |
| --- |

## Cochrane Library database search query

| ( [mh "Liver Diseases, Alcoholic"] OR (alcoholic AND ("liver disease*" OR steatosis OR fibrosis OR hepatitis OR cirrhosis))AND [mh Probiotics] OR probiotics:ti,ab,kw OR probiotic:ti,ab,kw)AND ( random*:ti,ab,kw OR placebo*:ti,ab,kw OR blind*:ti,ab,kw) |
| --- |

## 1.5. Scopus

| ((alcoholic AND ("liver disease*" OR steatosis OR fibrosis OR hepatitis OR cirrhosis))) AND ( probiotics OR probiotic) AND( random* OR placebo* OR blind*) |
| --- |

**Supplementary** **Figure 1. ALT**
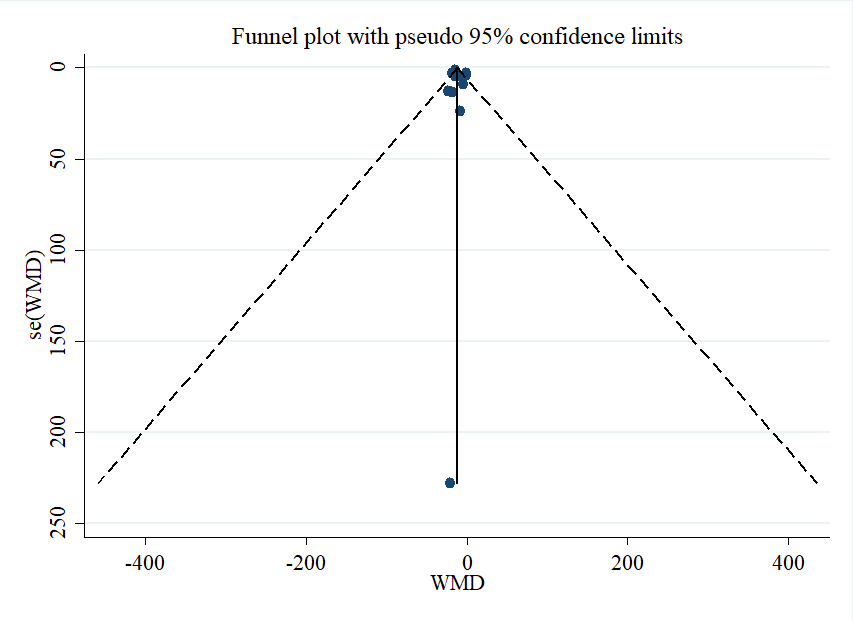


**Supplementary** **Figure 2. AST**


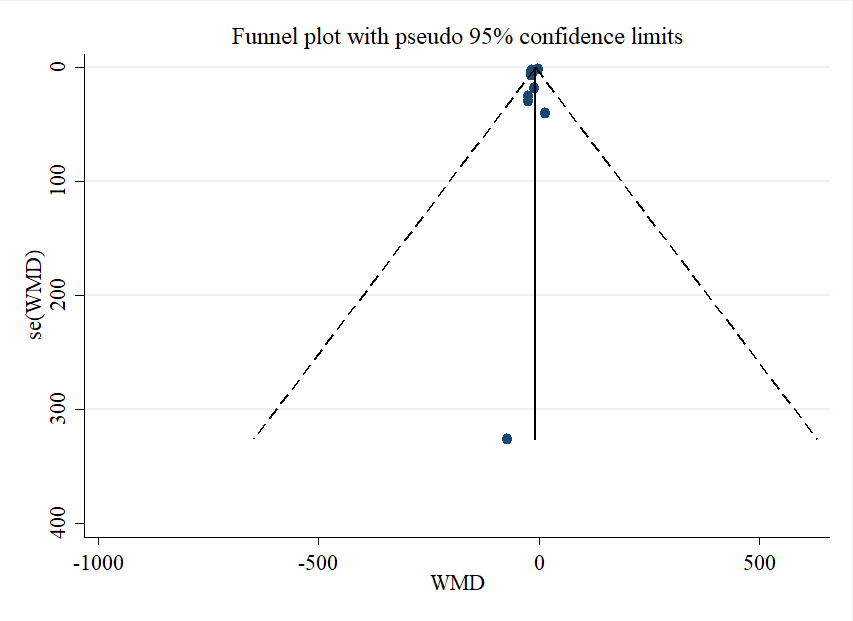


**Supplementary** **Figure 3. GGT** **
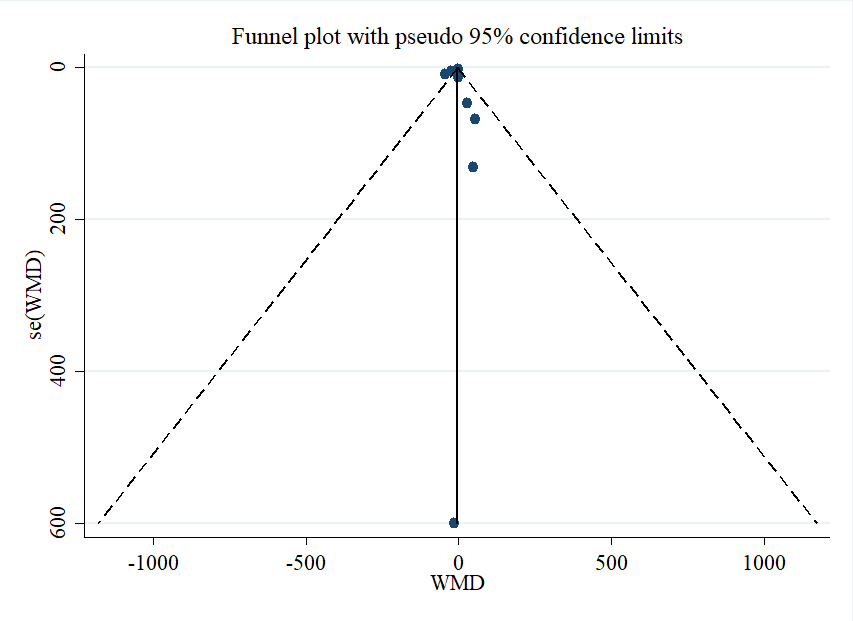
**

**Supplementary** **Figure 4. Trim and filled for GGT**
